# Pembrolizumab‐axitinib‐induced tumor lysis syndrome in a patient with metastatic renal cancer

**DOI:** 10.1002/ccr3.2737

**Published:** 2020-02-15

**Authors:** Manan Shah, Sanjay Jain, Temidayo Abe, Phani Keerthi Surapaneni, Kapil Bhatia

**Affiliations:** ^1^ Internal Medicine Morehouse School of Medicine Grady Memorial Hospital Atlanta Georgia; ^2^ Hematology‐Oncology Morehouse School of Medicine Grady Memorial Hospital Atlanta Georgia

**Keywords:** immunotherapy, pembrolizumab, prophylaxis, tumor lysis syndrome

## Abstract

Tumor lysis syndrome is uncommon in solid tumors but with the use of immunotherapy (checkpoint inhibitors) their incidence is increasing. Physicians need to take adequate precautions while treating patients with immunotherapy. The findings of our case report will help improve our current understanding of tumor lysis syndrome specially in solid tumors and will help in developing multidisciplinary treatment and prophylaxis strategies for this uncommon, but potentially fatal complication.

## Graphical abstract

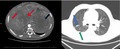

## 1 INTRODUCTION

Tumor lysis syndrome is an oncological emergency, which occurs as a result of breakdown of tumor cells after initiation of therapy leading to hyperkalemia, hyperuricemia, and release of cytokines in the body causing alterations in the normal cellular milieu.^1^, ^2^ More than half of the cases of tumor lysis are associated with hematological malignancies. However in the era of modern immunotherapy specially with tyrosine kinase inhibitors, their incidence is increasing.^3^, ^4^ Cairo and Bishop classification has been used to diagnose tumor lysis syndrome, which includes clinical and laboratory definitions.^5^ Laboratory Tumor lysis syndrome is defined as two or more of the following—uric acid above 8 mg/dL or 25% above base line, phosphate above 4 mg/dL or 25% above baseline and calcium below 7 mg/dL. Clinical tumor lysis syndrome is defined as the above plus one or more including seizure, raised creatinine, cardiac arrhythmias, or sudden death. Overall mortality can be as high as 79%.

## 2 CASE SUMMARY

**Figure 1:**
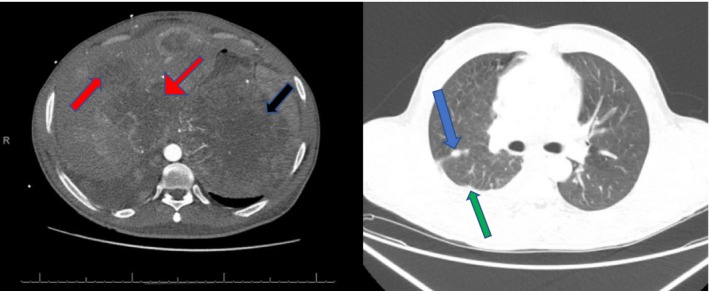
CT images showing lung metastasis (blue arrows), pleural‐based metastatic nodule (green arrow), large liver metastasis (red arrows), and a large approximately 10 × 9 cm left renal mass (black arrows)

A 37‐year‐old woman with a past medical history of hypertension, biopsy‐confirmed metastatic (Figure 1) clear cell renal carcinoma (metastasis to lung and liver), started on pembrolizumab‐axitinib (200/5 mg) 8 days ago presents from the outpatient cancer center complaining of fatigue and palpitations. On presentation, vital signs were blood pressure 98/70 mm Hg, pulse 118 bpm, respiratory rate 22, and temperature 98.6 F. Physical examination was significant for a nonobese female in acute distress, tachycardic with mild abdominal tenderness. Laboratory findings revealed potassium of 6.5 mg/dL, uric acid of 11.2 mg/dL, serum calcium of 8.8 mg/dL and serum creatinine of 1.5 mg/dL. Prechemotherapy laboratories were potassium 4.2 mg/dL, uric acid of 6.3 mg/dL, and calcium of 10 mg/dL (Table 1). EKG revealed sinus tachycardia with peaked T waves, and chest X‐ray was normal. The patient was admitted to the intensive care unit due to concern for tumor lysis syndrome. She was started on intravenous fluids, calcium gluconate, allopurinol, and insulin drip for hyperkalemia.

**Table 1:** Depicting laboratories before and after initiation of treatment

		On day of admission	Before treatment
Potassium‐serum	Latest ref range: 3.4‐5.1 meq/L	6.5 (HH)	4.2
Chloride‐serum	Latest ref range: 101‐111 meq/L	96 (L)	100
CO_2_ content‐serum	Latest ref range: 22‐32 mmol/L	29	28
Anion gap	Latest ref range: 1‐13 mmol/L	12	10
Glucose	Latest ref range: 70‐125 mg/dL	90	85
Urea nitrogen‐serum	Latest ref range: 8‐22 mg/dL	51 (H)	23
Creatinine	Latest ref range: 0.7‐1.2 mg/dL	1.5 (H)	0.9
Glomerular filtration rate	Latest ref range: >60 mL/min/1.73 mE2	>60	>60
Osmo, calculated	Latest ref range: 275‐300 mOsm/kg	287	295
Protein, total‐serum	Latest ref range: 6.0‐8.3 g/dL	8.4 (H)	6.6
Albumin, BCG‐serum	Latest ref range: 3.5‐5.0 g/dL	3.9	4.0
Calcium, albumin adjusted	Latest ref range: 8.9‐10.3 mg/dL	8.8	10
Calcium, total serum	Latest ref range: 8.9‐10.3 mg/dL	8.8	
Bilirubin, total‐serum	Latest ref range: 0.3‐1.6 mg/dL	1.6	1.5
Bilirubin, direct‐serum	Latest ref range: ≤0.5 mg/dL	0.5	0.5
AST (SGOT)	Latest ref range: 10‐42 U/L	40	38
ALT (SGPT)	Latest ref range: 17‐63 IU/L	74 (H)	60
Alkaline phosphatase serum	Latest ref range: 38‐126 IU/L	506 (H)	347
Uric acid	Latest ref range F‐3.4‐70 mg/dL	11	6.3

On the second day of admission, uric acid was 7.0 mg/dL, potassium 5.2 mg/dL, and creatinine at 1.5 mg/dL. She became short of breath and hypoxic. Oxygen saturation decreased to 86% on room air, and respiratory rate was 26 bpm. Follow‐up chest X‐ray revealed a diffuse infiltrate in the lungs concerning for acute respiratory distress syndrome (ARDS) and CT scan to rule out pulmonary embolism was negative. She was subsequently intubated and stabilized on mechanical ventilatory support.

By day 3, her laboratory findings revealed normal sodium, potassium, and uric acid levels. Her creatinine level was around 1.7 mg/dL. However, she continued to require high ventilatory support, developed a sudden cardiac arrest, and subsequently passed away. The cause of her death was attributed to ARDS.

## 3 DISCUSSION

We describe a patient with metastatic renal cell carcinoma started on pembrolizumab‐axitinib‐based therapy who developed tumor lysis syndrome within 8 days of initiation of therapy. To our knowledge, this is one of the fewer descriptions of this combination causing tumor lysis syndrome. Pembrolizumab is a anti‐PD‐1 drug, and axitinib is a tyrosine kinase inhibitor affecting VEGF receptors 1,2, and 3. It is believed that check point inhibitors like pembrolizumab lead to activation of T‐cell‐mediated cytokines destruction of tumor cells, thereby causing all the parametric changes.^6^, ^7^

Pembrolizumab‐Axitinib has been recently approved by FDA as the first‐line treatment for advanced and metastatic renal cell carcinoma. Approval was based on the Keystone‐426 trial, a randomized, multicentric trial. The trial demonstrated a statistically significant improvement in overall survival in patients treated with the above combination versus those treated with sunitinib.^8^ Pembrolizumab has also been used in metastatic cervical, endometrial, squamous cell lung cancer, and melanomas.^9^ The major adverse reactions associated with pembrolizumab include peripheral edema, fatigue, headaches, hypokalemia, hypoglycemia, hypomagnesemia, and diarrhea.^9^, ^10^

Tumor lysis syndrome is an oncological emergency that is caused by massive lysis of tumor cells with release of potassium and phosphate into the systemic circulation. Although there is general consensus that tumor lysis syndrome represents a set of metabolic complications that arise from treatment of rapidly proliferating neoplasm, there have been few attempts to specifically define them. Cairo Bishop definition, proposed in 2004, has been widely used for defining tumor lysis syndrome. It also includes a grading system to identify the severity of tumor lysis syndrome.^11^

Cairo Bishop Definition includes two major categories which includes the following:

- Laboratory tumor lysis syndrome—Defined as any two or more abnormal serum values of the parameters mentioned below within 3 days before or 7 days after institution of chemotherapy (Table 2).
- Clinical tumor lysis syndrome—Defined as laboratory tumor lysis syndrome plus one or more of the following that was not directly or probably attributable to a therapeutic agent: increased serum creatinine, cardiac arrhythmias/sudden death, or a seizure.

**Table 2:** Cairo Bishop definition with laboratory parameters

	Value	Change from baseline
Uric acid	>8 mg/dL	>25% from baseline
Potassium	>6 meq/dL	>25% from baseline
Phosphorus	>4.5 mg/dL	>25% from baseline
Calcium	>7 mg/dL	>25% from baseline

Risk factors that need to be taken into consideration while identifying tumor lysis syndrome include—chemosensitivity of the tumor, burden of the disease which includes size more than 10 cm, bone marrow involvement and pretreatment hyperuricemia and hyperphosphatemia. An expert panel identified prophylaxis recommendations based on tumor lysis syndrome risk mentioned in detail (Table 3).^11^ Low‐risk patients can be managed with aggressive intravenous hydration with or without allopurinol, while rasburicase is recommended for high‐risk patients.

**Table 3:** Table showing tumor lysis syndrome risk categories for prophylaxis

Low risk	Intermediate risk	High risk
Most solid tumors	Neuroblastoma, germ cell tumor, small cell lung cancer	Burkitt's leukemia
Multiple myeloma	Plasma cell leukemia	Acute myeloid leukemia with a WBC count >100 × 10^9^/L
Chronic myeloid leukemia	Acute myeloid leukemia with a WBC count of 25‐100 × 10^9^/L	Acute lymphoblastic leukemia with a WBC count >100 × 10^9^/L
Chronic lymphoid leukemia	Adult T cell leukemia/Lymphoma	Stage III and IV lymphomas
Adult intermediate Non‐Hodgkin's lymphoma with normal HDL	Acute lymphoblastic leukemia with WBC count <100 × 10^9^/L	Renal dysfunction
		Preexisting hyperuricemia and hyperphosphatemia

Hematological malignancies are most frequently associated with tumor lysis syndrome. In a systemic review of 387 adult patients showed that Acute leukemias which includes Acute myeloblastic leukemia and Acute Lymphocytic leukemia constituted 27 and 19 percent of patients who were at risk or who developed tumor lysis syndrome.^12^ Solid tumors were present in <1%.^12^ Solid tumors associated with tumor lysis syndrome mainly include small cell lung carcinoma, germ cell tumors, medulloblastoma, neuroblastoma, and urothelial tumors.^12^ In a recent systematic review of tumor lysis syndrome in solid malignancies, GU cancer was present in approximately 7% of the cases, all the reported cases received chemotherapy.Overall mortality rate was around 70%.^13^ To improve our understanding, we reviewed cases of tumor lysis syndrome in patients with renal cell cancer receiving immunotherapy (Table 4), the mean age was 64 years, tumor lysis syndrome was seen between 2‐14 days of treatment. The most striking feature of this review was that all the cases including our case had fatal consequences, thereby showing a mortality rate of 100%.^7^

**Table 4:** Case reports depicting tumor lysis syndrome due to checkpoint inhibitors in renal tumors

Author	Age	Gender	Cancer	Liver metastasis	Drug	Time to tumor lysis syndrome	Rasburicase	Outcome
Brunnhoelzl and Wang 10	77	F	Renal urothelial	Yes	Atezolizumab	Day 14	No	Death
Herbst et al^14^	70	N/A	Renal urothelial	N/A	Atezolizumab	N/A	N/A	N/A
Sater HA et al^15^	74	M	Renal cell carcinoma	No	Nivolumab	Day 2	N/A	Death
Our case report	37	F	Renal cell carcinoma	Yes	Pembrolizumab	Day 8	No	Death

Despite the above preventive measures, 3%‐5% of the patients will develop tumor lysis syndrome, which include laboratory and/or clinical evidence of tumor lysis syndrome as defined by Cairo Bishop classification.^13^

Differentials that need to be considered or that can mimic tumor lysis syndrome include renal failure secondary to nephrotoxic drugs or acute tubular necrosis, medications like thiazides causing hyperuricemia, ACE inhibitors, and potassium‐sparing diuretics (spironolactone) causing hyperkalemia.

Unlike tumor lysis syndrome in hematological malignancies its cause in solid tumors is not well understood. The pattern of tumor lysis syndrome in solid tumors is variable. This may explain why the mortality associated with tumor lysis tumor is higher than in hematological malignancies.^12^, ^13^

The findings of our case report will help improve our current understanding of tumor lysis syndrome specially in solid tumors and will help in developing multidisciplinary treatment and prophylaxis strategies for this uncommon, but potentially fatal complication. The importance of being cautious while dealing with newer immunotherapy agents has been reiterated with this case report.

## 4 CONCLUSION

Our case is unique and possibly the first description of pembrolizumab causing tumor lysis syndrome. In addition to this, tumor lysis syndrome in solid tumors is uncommon, but with the increasing use of immunotherapy combinations physicians should pay attention to rising rates of tumor lysis syndrome in solid tumors which have potentially fatal consequences.

## CONFLICT OF INTEREST

None.

## **AUTHOR** **CONTRIBUTION**

MS and SJ**:** was involved in the conception and design of the work, data collection, drafting of the manuscript, critical revision of the manuscript, and final approval of the version to be published; TA, PS, and KB: reviewed literature and helped in revisions of the manuscript.
